# Long-Time Cooling before Cryopreservation Decreased Translocation of Phosphatidylserine (Ptd-L-Ser) in Human Ovarian Tissue

**DOI:** 10.1371/journal.pone.0129108

**Published:** 2015-06-17

**Authors:** Vladimir Isachenko, Plamen Todorov, Evgenia Isachenko, Gohar Rahimi, Andrey Tchorbanov, Nikolina Mihaylova, Iliyan Manoylov, Peter Mallmann, Markus Merzenich

**Affiliations:** 1 Research Group for Reproductive Medicine and IVF-Laboratory, CAM-Xenotransplantation Group, Department of Obstetrics and Genecology, Cologne University, Cologne, Germany; 2 Institute of Biology and Immunology of Reproduction, Sofia, Bulgaria; 3 Institute of Microbiology, Sofia, Bulgaria; 4 Praxisklinik Schoenhauserstrasse, Cologne, Germany; French Blood Institute, FRANCE

## Abstract

**Objectives:**

To translocation (externalization) of phosphatidylserine lead at least the five negative effects observed during cells cryopreservation: hypoxia, increasing of intracellular Ca^2+^, osmotic disruption of cellular membranes, generation of reactive oxygen species (ROS) and lipid peroxidation. The aim of this study was to test the intensiveness of the phosphatidylserine translocation immediately after thawing and after 45 d xenografting of human ovarian tissue, which was either frozen just after operative removal from patient or cooled before cryopreservation to 5°C for 24 h and then frozen.

**Materials and Methods:**

Ovarian fragments from twelve patients were divided into small pieces in form of cortex with medulla, and randomly divided into the following four groups. Pieces of Group 1 (n=30) were frozen immediately after operation, thawed and just after thawing their quality was analyzed. Group 2 pieces (n=30) after operation were cooled to 5°C for 24 h, then frozen after 24 h pre-cooling to 5°C, thawed and just after thawing their quality was analyzed. Group 3 pieces (n=30) were frozen immediately after operation without pre-cooling, thawed, transplanted to SCID mice and then, after 45 d of culture their quality was analyzed. Group 4 pieces (n=30) were frozen after 24 h pre-cooling to 5°C, thawed, transplanted to SCID mice and then, after 45 d their quality was analyzed. The effectiveness of the pre-freezing cooling of tissuewas evaluated by the development of follicles (histology) and by intensiveness of translocation of phosphatidylserine (FACS with FITC-Annexin V and Propidium Iodide).

**Results:**

For groups 1, 2, 3 and 4 the mean densities of follicles per 1 mm^3^ was 19.0, 20.2, 12.9, and 12.2, respectively (P_1-2, 3-4_ >0.1). For these groups, 99%, 98%, 88% and 90% preantral follicles, respectively were morphologically normal (P_1-2, 3-4_ >0.1). The FACS analysis showed significantly decreased intensiveness of translocation of phosphatidylserine after pre-cooling of frozen tissue (46.3% and 33.6% in Groups 2 and 4, respectively), in contrast with tissue frozen without pre-cooling (77.1% and 60.2 % in Groups 1 and 3, respectively, P_1, 3-2, 4_ <0.05).

**Conclusions:**

Long time (24 h) cooling of ovarian tissue to 5°C before cryopreservation decreased translocation of phosphatidylserine that evidences about increases the viability of the cells in the tissue after thawing.

## Introduction

Cancer is one of the major death causes in the world. In the USA alone a total of 1,658,370 new cancer cases and 589,430 cancer deaths are projected to occur in 2015 [1]. The overall incidence rate for cancer in children aged 14 years and younger increased by 0.6% per year between 1998 and 2007 [2].

A similar trend has been observed in Europe. The current estimate for 2010 for Germany relates to a total of approximately 204,000 cancer cases in women, and every year in Germany, around 800 girls under age 15 are diagnosed with cancer [3]. At the same time, increased survival rates were observed for all categories of childhood cancers studied, with the extent and temporal pace of the increases varying by diagnosis [4].

Due to the increasing of effectiveness of cancer treatments and positive long-term prognosis for young women, the problem of post-cancer infertility plays a significant role because chemotherapy can be gonadotoxic and lead to the functional death of ovaries. Cryopreservation of ovarian tissue before cancer therapy with re-implantation after convalescence is the potential key solution of this problem [5, 6].

Several cases reporting restoration of ovarian function after re-implantation of ovarian cortex in patients with premature ovarian failure after cryopreservation of ovarian tissue before the cancer treatment have been published since 1998. Now pregnancies and baby-born after orthotropic retransplantation of frozen ovarian tissue were reported [7]. Baby born after heterotopic transplantation are absent [8], even this direction of reproductive medicine is perspective: several studies have reported restoration of ovarian function as well as potential fertility after heterotopic transplantation in monkeys and humans [9].

Phosphatidylserine is a phospholipid component of membrane which plays a key role in cell cycle signaling, specifically in relationship to necrosis and apoptosis. When a cell affected by some negative factors, phosphatidylserine is no longer restricted to the intracellular side of membrane and translocated to the extracellular surface of the cell. This is they act as a signal for macrophages to engulf the cells [10]. At least five negative effects observed during cells cryopreservation: hypoxia, increasing of intracellular Ca^2+^, osmotic disruption of cellular membranes, generation of reactive oxygen species (ROS) and lipid peroxidation. Each from these factors can lead to translocation of phosphatidylserine.

In fact, data about the phosphatidylserine translocation in cryopreserved human ovarian tissue is limited.

A positive effect on the future development of cells, which have been cooled to low suprazero temperatures and then thawed, has been observed before and is not new. At present, the suprazero temperature (usually from 0°C to 7°C) storage method is a widely used technology in organ preservation. Low temperature retards cellular metabolism and thus reduces cellular oxygen demand and consumption. This furnished an approach to reduce tissue autolysis without the need for vascular perfusion. The absence of a negative effect of hypothermic storage on domestic cat [11] and rat [12] ovaries has been noted. It was reported about positive effect of cooling on rat hypothalamus [13].

The aim of this study was to test the intensiveness of the phosphatidylserine translocation immediately after thawing and after 45 d xenografting of human ovarian tissue, which was either frozen just after operative removal from patient or cooled before cryopreservation to 5°C for 24 h and then frozen.

## Materials and Methods

Full details of the study described in this article were approved by the Ethics Boards of Universities Cologne (Applications 99184 and 13–147). Manipulations with SCID mice were approved by the Animal Care Commission at the Institute of Microbiology, Sofia, Bulgaria.

Written informed consents were obtained from all participants aged 18 and over involved in our study.

On the behalf of the patients under the age of 18 written consents were obtained from the next of kin.

Except where otherwise stated, all chemicals were obtained from Sigma (Sigma Chemical Co., St. Louis, MO, USA).

### Tissue collection, dissection, and distribution into groups

Informed consent was obtained from 12 patients aged between 15 and 38 (25.2±4.1) years. According to approved protocol 10% of ovarian tissue was used for patient-oriented research.

The basal medium used for manipulation of tissues (transport and dissection) was Leibovitz L-15 with 5% Dextran Serum Substitute (Irvine Scientific, Santa Ana, CA, USA), referred to below as ‘basal medium’.

Fresh ovarian tissue fragments were transported from the surgical room to the laboratory within 20 min with temperature maintained at 32–34°C. Using tweezers and scalpel No 22, ovarian fragments were dissected into small pieces (1.5–2.0 x 1.0–1.2 x 1.0–1.2 mm) and cryopreserved as described below. The pieces were randomly divided into four groups:

Group 1 (n = 30): pieces were frozen immediately after operation, thawed and just after thawing their quality was analyzed.
Group 2 (n = 30): pieces after operation were cooled to 5°C for 24 h, then frozen, thawed and just after thawing their quality was analyzed.
Group 3 (n = 30): pieces were frozen immediately after operation, without pre-cooling, thawed, transplanted to SCID mice and then, after 45 d of culture their quality was analyzed.
Group 4 (n = 30) pieces were frozen after 24 h pre-cooling to 5°C, thawed, transplanted to SCID mice and then, after 45 d their quality was analyzed.

Thirty, corresponding to two pieces per mouse in each experimental groups, 120 pieces were used to determine the quality of follicles and the degree of phosphatidylserine translocation.

### Tissue cryopreservation (freezing and thawing)

Pieces of ovarian tissue were placed at room temperature in 20 ml freezing medium composed of basal medium supplemented with 6% dimethyl sulfoxide, 6% ethylene glycol and 0.15 M sucrose. Then pieces were put into a standard 5-ml cryo-vials (Thermo Fisher Scientific, Rochester, NY, USA) previously filled by freezing medium and frozen in a IceCube 14S freezer freezer (SyLab, Neupurkersdorf, Austria). The cryopreservation programm was as follows:(i) the starting temperature was -6°C; (ii) samples were cooled from -6°C to -34°C at a rate of -0.3°C/min; (iii) at -34°C cryo-vials were plunged into liquid nitrogen. The freezing protocol for cryopreservation of this ovarian tissue included an auto-seeding step at -6°C.

The procedure of thawing was achieved by holding the vial for 30 s at room temperature followed by immersion in a 100°C (boiling) water bath for 60 s, and expelling the contents of the straw into the solution for the removal of cryoprotectants. The exposure time in the boiling water was visually controlled by the presence of ice in the medium; as soon as the ice was 2 to 1 mm apex, the straw was removed from the boiling water, at which point the final temperature of the medium was between 4 and 10°C. Within 5–10 seconds after thawing, the pieces from the cryo-vials were expelled into 10 ml thawing solution (basal medium containing 0.5 M sucrose) in a 100 ml specimen container (Sarstedt, Nuembrecht, Germany). The stepwise dilution of cryoprotectants was achieved using the same principle as that used for saturation by ethylene glycol [14]. The container was placed on a shaker and continuously agitated with 200 osc/min for 15 min at room temperature. Stepwise rehydration of the tissue pieces for 30 min at room temperature was also performed using the same ‘dropping’ methodology: slow addition of basal medium (see above) to the solution of sucrose with ovarian pieces. For ‘dropping’, we used 50 ml of basal medium in a 50 ml tube (Greiner Bio-One GmbH, Frickenhausen, Germany). The final sucrose concentration was 0.083M, resulting in almost isotonic conditions. Finally, the pieces were washed thrice each in basal medium for 10 min, and grafted to SCID-mice.

### Xenografting of ovarian tissue

Thirty female 7 weeks old SCID (BALB/c) mice were obtained from Harlan Farm (Blackthorn, UK). The animals had access to food and water ad libitum under 12 h light, 12 h dark conditions. They were kept under specific-pathogen-free (SPF) conditions.

**Fig 1:**
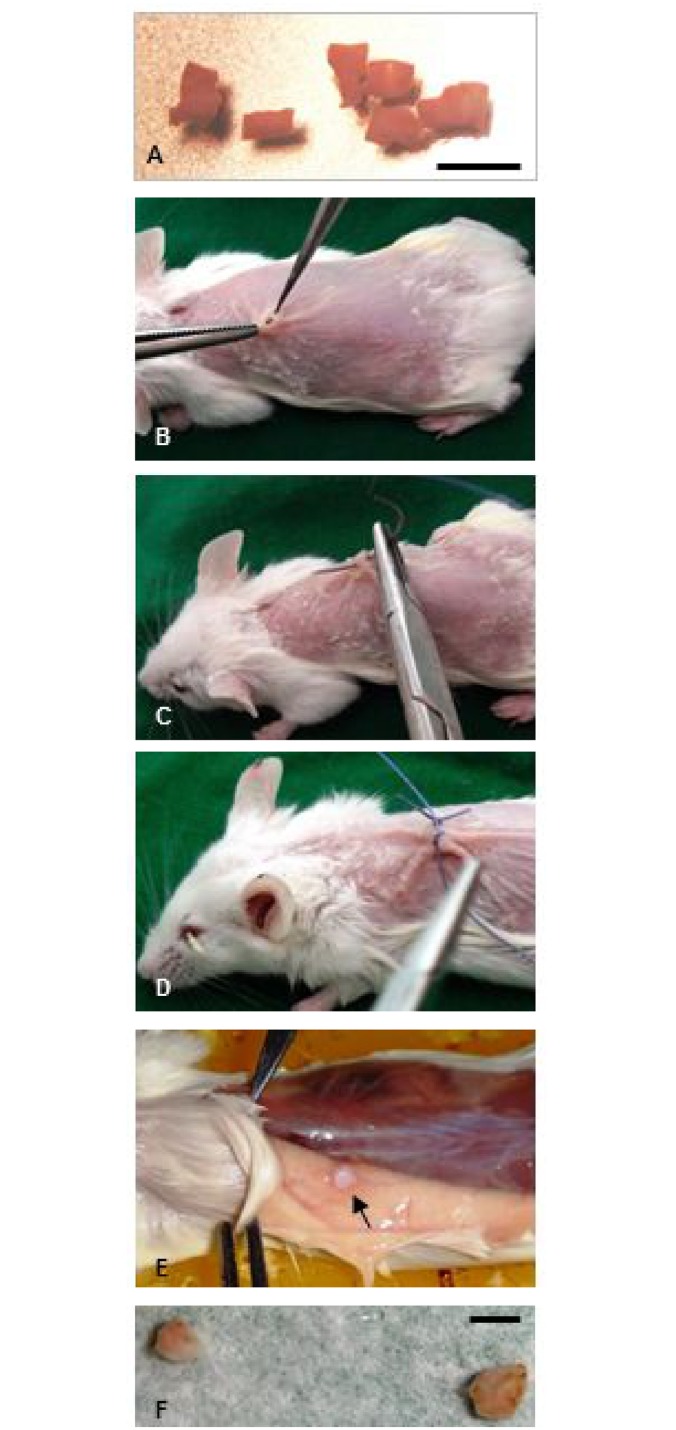
Xenografting of cryopreserved human ovarian tissue. (A) Ovarian pieces after thawing, (B, C, D) Transplantation of these pieces, (E, F) Ovarian pieces after 45 d xenografting. Scale bar: 2 mm.

Immediately after thawing, the quality of tissue was analyzed (Groups 1 and 2) or ovarian pieces were transplanted to SCID mice (Groups 3 and 4) as described early [15–17] (Fig 1). During surgery, the mice were kept on a warming plate and the incision site was disinfected and covered with a sterile towel. For operation the combination of anesthetic Rompun 2% (Bayer Vetal Leverkusen, Germany) and Ketanest 50 mg/ml (Parke-Davis Freiburg, Germany) was used. In each mouse the tissue pieces were grafted dorsolateral on the right and left side of the vertebral column. Every second day the mice were injected with 1.0 IU of recombinant FSH (Gonal-F, Laboratories Serono S.A., Aubonne, Switzerland). The animals were killed by cervical dislocation on the 45th day after transplantation of ovarian tissue.

Immediately after thawing or after 45 d xenotransplantation, each from 120 recovered grafts was dissected into two particles: the one part was processed for measurement of phosphatidylserine translocation and the second one fixed for histological investigation.

### Phosphatidylserine translocation: flow cytometry

The human tissue was isolated from the mice and replaced to PBS with 10% fetal calf serum (FCS). Later, this tissue was incubated in GM 501 Collagenase (Gynemed GmbH, Lensahn, Germany) for 1h at 37°C with shaking. The reaction was stopped by adding of ice cold PBS-FCS, followed by mechanical disruption of the tissue through 20G syringe needle and passing through 70 μm filter on ice. The cells were centrifuged at 1000 RPM at 4°C for 10 min in PBS-FCS and suspended in 1 ml PBS-FCS.

The isolated cells were cultured in complete RPMI 1640 (Roswell Park Memorial Institute medium, Gibco, Gaithersburg, MD, USA) containing 10% FCS, 4 mM L-glutamine, 50 μM 2-mercaptoethanol and antibiotics for 1 h at 37°C in 5% CO_2_. Then cells were collected, washed and stained with FITC-Annexin V apoptosis detection Kit I (BD Biosciences, San Diego, CA, USA), containing Propidium Iodide (PI) as DNA-binding dye according manufacturer instruction. The apoptosis of gated cells was analyzed within 15 min using flow cytometry (BD LSR II flow cytometer). The rate of phosphatidylserine translocation as detector (high level of probability, P<0.05) of early apoptosis (FITC-Annexin V positive, PI negative), late apoptosis and dead cells (FITC-Annexin V positive, PI positive), necrosis (FITC-Annexin V negative, PI positive) as well as viable cells (both FITC-Annexin V and PI negative) were measured.

### Histology of follicles

For histological investigation, the cultured tissue pieces were fixed in Bouin’s solution, embedded in paraffin wax, serially sectioned at 4 μm, stained with hematoxylin/eosin, and analyzed under a microscope (x400, Olympus Co, Tokyo, Japan).

The number of viable and damaged follicles was counted. Before counting of follicles, sections were coded and scored ‘‘blind”. To avoid over-counting of the same follicles, only the section with a visible oocyte nucleus was taken into account. Two types of preantral follicles were evaluated: 1) primordial follicles composed of an oocyte surrounded by a layer of flattened follicular cells and 2) primary and secondary follicles which are similar to primordial follicles, but in which the oocyte is surrounded by one to two layers of spheroid granulosa cells.

The quality of follicles was graded on the scale from one to three. A follicle of grade 1 is spherical in shape and contains a spherical oocyte which is surrounded by an even distribution of granulosa cells and has a homogenous cytoplasm and slightly granulated nucleus, with condensed chromatin in the form of a dense spherical structure detectable in the center of the nucleus. A follicle of grade 2 has similar characteristics, but the oocyte is without condensed chromatin within the nucleus and is often irregular in shape; the surrounding granulosa cells can be flat and pulled away from the edge of the follicle. A follicle of grade 3 contains a misshapen oocyte with or without nuclear vacuolation; theca and granulosa cells are separated from the edge of the follicle and the partly or fully disrupted granulosa have pyknotic nuclei. Follicles of grades 1 and 2 were denoted as normal and those of grade 3 were denoted as degenerated. Examples of the different follicular degenerations can be observed elsewhere (for example, see [14, 18–22].

### Statistical analysis

Rate of in cells of tissue after treatment was evaluated by ANOVA. Orthogonal contrasts were used to separate main effects. The level of statistical significance was set at a P <0.05.

## Results

Histological examination just after thawing as well as after 45 d development showed that only preantral (primordial, primary and secondary) follicles were viable. All the antral follicles in these two groups were degenerated and hence were not counted.

**Fig 2:**
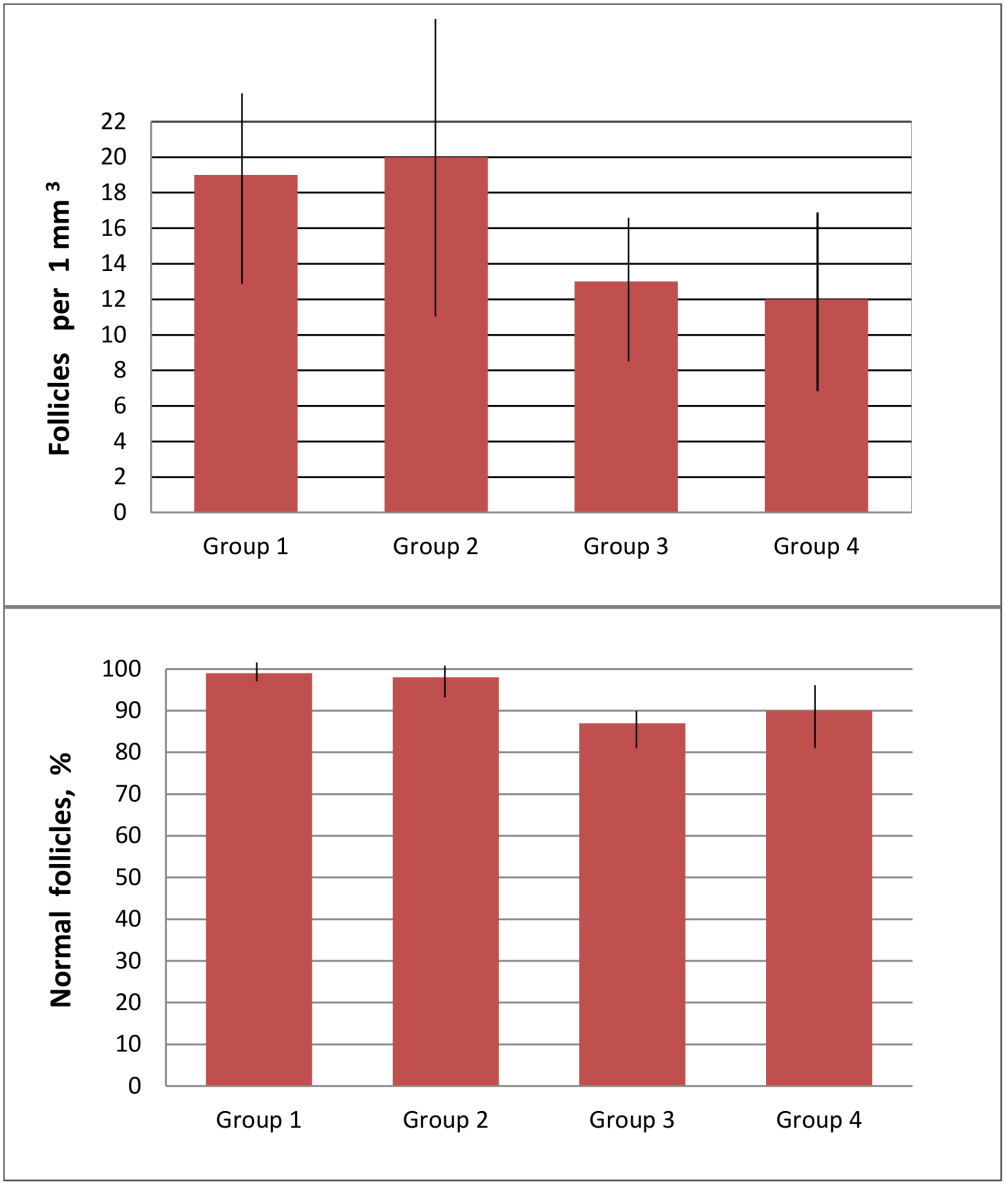
Effect of cooling on the quality of follicles (expressed as quantity and normality of follicles). Group 1: freezing of tissue without pre-cooling, then thawing and evaluation of their quality. Group 2: freezing of tissue after 24h pre-cooling to 5°C, then thawing and evaluation of their quality. Group 3: freezing of tissue without pre-cooling, then thawing, xenografting and evaluation of their quality. Group 4: freezing of tissue without pre-cooling, then thawing, xenografting and evaluation of their quality. No statistical differences between respective groups (Group 1 *vs* Group 2 and Group 3 *vs* Group 4 (P>0.1).

For groups 1, 2, 3 and 4 the mean densities of follicles per 1 mm^3^ was 19.0±5.6, 20.2±8.1, 12.9±4.4, and 12.2±5.1, respectively (P_1-2, 3-4_ >0.1). For these groups, respectively 99.0±2.1%, 98.0±3.0%, 87.7±4.1% and 90.0±5.7% preantral follicles were morphologically normal (P_1-2, 3-4_ >0.1) (Fig 2).

**Fig 3:**
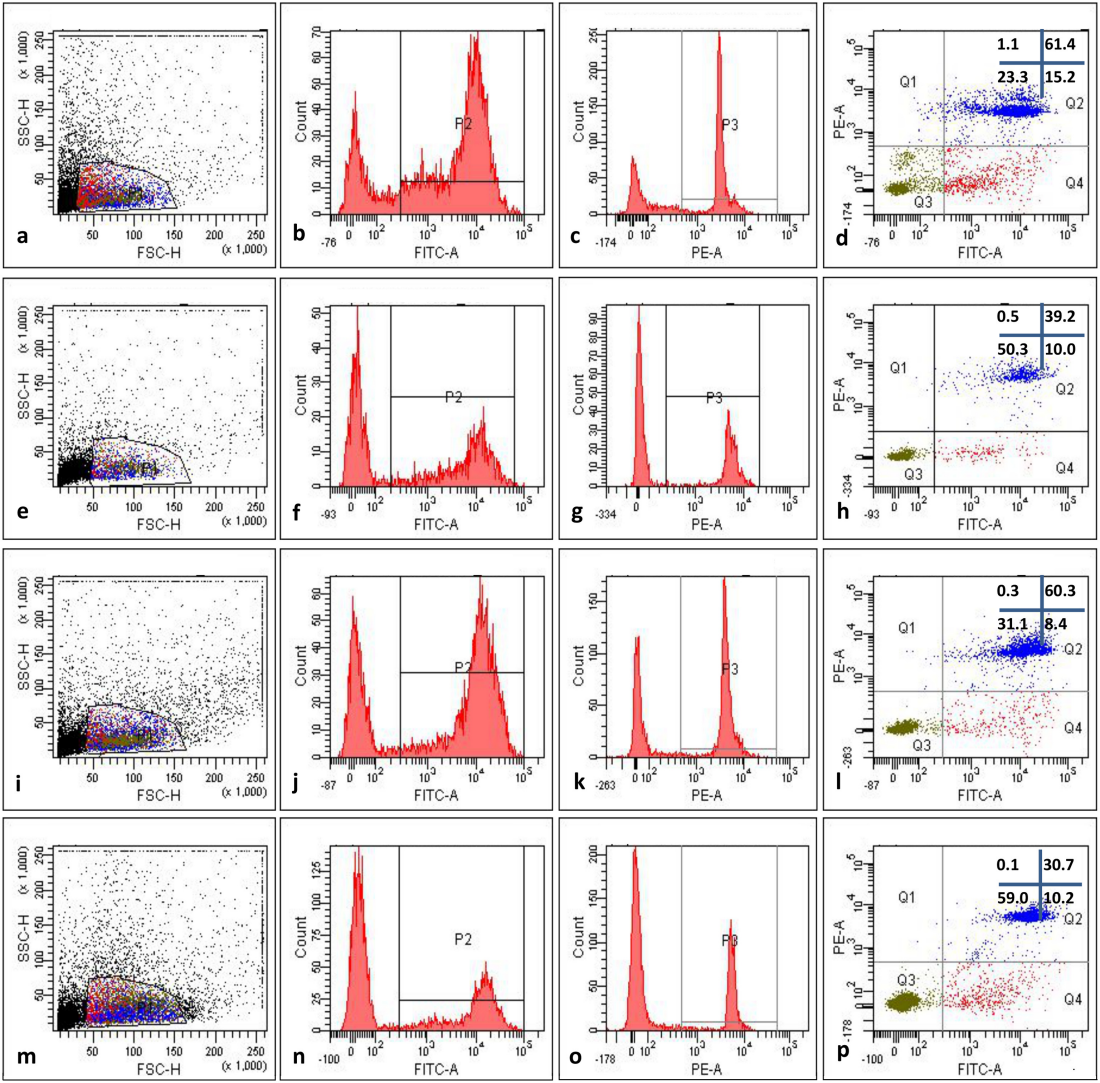
Translocation of phosphatidylserine in ovarian tissue pre-cooled to 5°C for 24 h before the freezing and then xenografted in SCID mice: representative example of one experiment. (a, b, c, d) Group 1: pieces were frozen immediately after operation, thawed and just after thawing their quality was FACS analyzed, (e, f, g, h) Group 2: pieces after operation were cooled to 5°C for 24 h, then frozen, thawed and just after thawing their quality was FACS analyzed, (i, j, k, l), pieces were frozen immediately after operation, without pre-cooling, thawed, transplanted to SCID mice and then, after 45 d of culture their quality was FACS analyzed. (m, n, o, p) pieces were frozen after 24 h pre-cooling to 5°C, thawed, transplanted to SCID mice and then, after 45 d their quality was FACS analyzed, (a, e, i, m) forward and scatter dot plot used to select the interest population, (c, g, k, o) histograms displaying fluorescence of PE channel, used to measure fluorescence intensity of Propidium Iodide (PI), (d, h, i, p) dot plot analysis of FITC-Annexin V and PE channels, (Q1) cells negative to Annexin V (FITC A) and positive to PI (could indicate necrotic cells), (Q2) cells positive to both Annexin V and PI (could indicate late apoptotic stage), (Q3) cells negative for both Annexin V and PI (could indicate viable cells), (Q4) cells positive to FITC-Annexin V and negative to PI (could indicate early apoptotic state).

In Group 1 cells (ovarian tissue, which was frozen just after operative removal, then thawed and their quality was analyzed) 14.3±0.6% of cells were in early apoptotic state (FITC-Annexin V positive, PI negative), while a 62.8±4.9% of cells showed characteristics of late apoptotic state or dead cells (FITC-Annexin V positive, PI positive). In this group 22.8±3.7% were viable cells (both FITC-Annexin V and PI negative) and 1.0±0.1% corresponded to necrotic cells (FITC-Annexin V negative, PI positive) (Fig 3).

In Group 2 cells (ovarian tissue, which was frozen after 24 h cooling to +5°C just, then thawed and their quality was analyzed) 9.1± 1.7 of cells were in early apoptotic state (FITC-Annexin V positive, PI negative), while a 37.2±3.7% of cells showed characteristics of late apoptotic state or dead cells (FITC-Annexin V positive, PI positive). In this group 50.4±4.7% were viable cells (both FITC-Annexin V and PI negative) and 3.3±0.2% corresponded to necrotic cells (Fig 3).

In Group 3 cells (ovarian tissue, which was frozen just after operative removal, then thawed, transplanted to SCID mice and their quality was analyzed) 7.4±0.7% of cells were in early apoptotic state (FITC-Annexin V positive, PI negative), while a 52.8±4.8% of cells showed characteristics of late apoptotic state or dead cells. In this group 39.5±4.1% were viable cells (both FITC-Annexin V and PI negative) and 0.5±0.0% corresponded to necrotic cells (Fig 3).

In Group 4 cells (ovarian tissue, which was cooled after operation to 5°C for 24 h, frozen and then thawed, transplanted to SCID mice and their quality was analized) it was observed 9.2±2.0% cells in early apoptotic state and 24.4±1.9% cells late apoptotic state. In this group 66.2% cells were viable and 0.2±0.0% of cells corresponded to necrotic ones (Fig 3).

## Discussion

### Phosphatidylserine as detector not only of apoptosis

Phosphatidylserine is a phospholipid component of membrane which plays a key role in cell cycle signaling, specifically in relationship to necrosis and apoptosis.

Phosphatidylserine is normally located on the inner side of the cell membrane. However, when a cell affected by some negative factors, phosphatidylserine is no longer restricted to the intracellular side of membrane and translocated to the extracellular surface of the cell, they act as a signal for macrophages to engulf the cells [10].

In discussion of our results we specially try to avoid the word "apoptosis" when we write about translocation of phosphatidylserine after cryopreservation of cells. In fact, from the beginning of 1990s it was concluded that the translocation of phosphatidylserine can play the role of detector of apoptosis [23]. However, later it was established that this process of phosphatidylserine translocation may be reversible and occurs also in T-cell activation, without cell death [24].

Apoptotis is measured after labeling of cellular DNA with different dyes or by using terminal deoxynucleotide transferase assay. Commercial kits for apoptosis detection are based on the principle of interaction between Annexin V and phosphatidylserine located on the cell surface. Dead cells are distinguished from live ones by a second dye such as Propidium Iodide. However, necrotic cells can also express phosphatidylserine and number of authors write that there is a need of more precise discrimination between apoptotic and necrotic cells [23, 25–29].

It was concluded that phosphatidylserine translocationon the outer leaflet of the plasma membrane, are indeed less universal and more context dependent than previously thought [30–34]. Galluzzi et al. [33] written that as a caveat to the use of phosphatidylserine alone as a marker of early apoptosis, it should be noted that (1) if plasma membranes are permeabilized (as during late apoptosis or early necrosis) Annexin V can bind to intracellular phosphatidylserine; (2) phosphatidylserine translocation can prepare cells for phagocytic removal independently of apoptosis [35] and that (3) phosphatidylserine translocation can be compromised in cells in which autophagy is impaired [36].

As noted [37], a ‘specific’ cell death-related phenomenon may occur along with the execution of another cell death mode. For instance, excessive generation of reactive oxygen species (ROS) and reactive nitrogen species has been associated with several cases of apoptosis [38–40]. Increased membrane permeability is a signal for early apoptosis both in somatic cells [41].

It is also of interest to note that one from specific characteristics of cancer cells, development of which directly connected with effect(s) of some negative factor(s) on the normal cells [42].

Taking into account all mentioned above, our point of view was formulated as follows: we evaluate the translocation of phosphatidylserine after respective cryo-treatment not as detector of apoptotic changes in cells but brightly, as detector of viability of these cells after treatment. By our opinion, for evaluation of technology of cryopreservation, which includes in our case the pre-freezing treatment by cold, the question "what was happened with frozen cells after thawing and xenografting, apoptosis or necrosis?" place not a first role. By interpretation of our results we evaluate an increased translocation of phosphatidylserine as reaction of cells on cryobiological treatment.

### Phosphatidylserine as detector of cryo-damages

At least five negative effects observed during cells cryopreservation: hypoxia, increasing of intracellular Ca^2+^, osmotic (relatively fine) disruption of cellular membranes, generation of reactive oxygen species (ROS) and lipid peroxidation. Each from these factors can lead to translocation of phosphatidylserine.

It has been noted that during cryopreservation a cell initiates intracellular apoptotic or necrotic signaling in response to a stress. The binding of nuclear receptors by heat, hypoxia [43] and increased intracellular calcium concentration [44] for example, by damage to the membrane, can all trigger the release of intracellular apoptotic signals by a damaged cell. Apoptotic proteins that target mitochondria affect them in different ways. They may cause mitochondrial swelling through the formation of membrane pores, or they may increase the permeability of the mitochondrial membrane and cause apoptotic effectors to leak out [43].

Apoptosis is a major cause of sperm damage during cryopreservation [45].

It was established for human ovarian tissue that the percentage of apoptotic follicles was significantly higher in cryopreserved compared to the untransplanted tissue at baseline (15% vs. 4%), indicating that cryopreservation process itself induces apoptotic process in follicles [46].

And if detection of apoptosis in cells after respective treatment (for example, by TUNEL-test) indirectly allows us to say about translocation of phosphatidylserine, the data presented below evidence that a cryopreservation treatment leads just to this process.

Cryopreserved blood cells have high level of phosphatidylserine translocation and contained significantly more phosphatidylserine-positive microparticles than liquid-stored cells [47, 48].

It has been reported that cryopreservation of human spermatozoa produces chemical oxidative stress [49] and an apoptosis-like phenomenon [30] in spermatozoa which may cause various structural alterations of spermatozoa such as the disturbance of the plasma membrane, notably characterized by an translocation of phosphatidylserine. Authors concluded that by this reason, the percentages of live cells with phosphatidylserine translocation increased significantly after thawing [50, 51].

Data about translocation of phosphatidylserine in cells of human ovarian tissue are limited.

### Cells and positive effect of cooling

A positive effect of cooling of cells to low supra-zero temperatures on their future development after re-warming has been observed before and is not new.

It was shown that the acclimation of the warm-blooded rat to cold stimulates mitosis indirectly in cells capable of division because it stimulates directly the mitotic activity in mouse and human cells cultured and adapted to the cold *in vitro*. In situ hybridization analysis of hypothalamic tissue showed that cold exposure causes a two-fold increase in the total number of neurones expressing thyrotrophin-releasing hormone mRNA in the paraventricular nucleus [13].

In addition, our previous results showed good survival of bovine trophoblastic fragments that had been subjected to chilling for 48 h at 4°C. The survival/formation of vesicles in these fragments was not different from that of the untreated controls (98% and 98%, respectively) [52].

The exposure of human ovarian tissue to low positive temperatures of up to 26 h does not inhibit the development of follicles during subsequent *in vitro* culture. Compared with the untreated controls, the number of primordial follicles in all treatment groups significantly decreased owing to development to the advanced stages [53]. The results of an investigation by Yin et al. [12], who found that, although ovaries showed fewer follicles, hypothermic storage of rat ovary at 4°C for 24 h did not disrupt ovarian function, does not support our results. This could be explained by the species-specific sensitivity of ovarian tissue to hypothermia.

Interesting investigations were performed by Wood et al. [11]. The influence of long-term hypothermic storage of whole domestic cat ovary for 48 h at 4°C on follicle-oocyte atresia and temporal taphonomy was investigated. It was found that the highest (but statistically insignificant) degeneration rate of follicles occurred at 48 h, with inhibition of taphonomy. Our data support these results.

In conclusion, it appears that cooling of ovarian tissue to 5°C for 24 h before cryopreservation decreased translocation of phosphatidylserine that evidences about increases the viability of the cells in the tissue after thawing.
