# Correction: Long-Time Cooling before Cryopreservation Decreased Translocation of Phosphatidylserine (Ptd-L-Ser) in Human Ovarian Tissue

**DOI:** 10.1371/journal.pone.0212961

**Published:** 2019-02-22

**Authors:** Vladimir Isachenko, Plamen Todorov, Evgenia Isachenko, Gohar Rahimi, Andrey Tchorbanov, Nikolina Mihaylova, Iliyan Manoylov, Peter Mallmann, Markus Merzenich

There are errors in the Data Availability Statement; the raw data underlying Figs 2 and 3 are not provided within the paper. The authors have provided the summary level data underlying Fig 2 and the raw data underlying Fig 3 as Supporting Information files below.

## Supporting information

S1 File Summary level data underlying Fig 2. (XLSX)

S2 File Raw data underlying Fig 3. (ZIP)
